# Healthcare transition practices of occupational therapists in South African public healthcare

**DOI:** 10.4102/ajod.v13i0.1413

**Published:** 2024-08-29

**Authors:** Ilhaam Hoosen, Fiona Breytenbach, Janine van der Linde

**Affiliations:** 1 Department of Occupational Therapy, Faculty of Health Sciences, School of Therapeutic Sciences, University of the Witwatersrand, Johannesburg, South Africa

**Keywords:** healthcare transition, adolescence, occupational therapy, paediatric to adult healthcare, HCT

## Abstract

**Background:**

Healthcare transition (HCT), the process of transitioning an adolescent from paediatric- to adult-oriented care, is vital for improving the long-term health of adolescents with chronic conditions. The role of occupational therapy in HCT has not been well-researched. Effective HCT practices are necessary to ensure that adolescents have access to coordinated, optimal and uninterrupted occupational therapy services throughout this period of development.

**Objectives:**

This study describes occupational therapists’ self-perceived knowledge of HCT within the context of South African public health facilities, the HCT practices used, and the factors that promote or hinder the success of HCT within this context.

**Method:**

The study utilised a quantitative, non-experimental and descriptive cross-sectional design. Simple convenience and snowball sampling were used to recruit participants via professional databases and social media forums. An online survey was used to collect data. Descriptive statistics and simple content analysis were used to analyse the information.

**Results:**

This study identifies limitations in the knowledge and practical implementation of HCT within South African occupational therapy practice. Healthcare transition is characterised by inadequate use of policies, insufficient transition preparation and poor outcome measurements.

**Conclusion:**

There is a need for the development of training programmes and practice guidelines to optimise and support HCT implementation within South African occupational therapy practice.

**Contribution:**

This study provides novel data on HCT practices utilised by occupational therapists in South African public health facilities. This study has potential use for the development of effective HCT programmes that can improve the functional outcomes of South African adolescents.

## Introduction

Healthcare transition (HCT) is a critical, but often neglected, process that is integral to ensuring the continuity of healthcare services between childhood and adulthood. Healthcare transition is the process of transferring an adolescent from child-oriented to adult-oriented healthcare in an uninterrupted, developmentally appropriate and coordinated manner (Blum et al. 1993; Hasegawa & Gleeson 2018). Adolescents with chronic illnesses or functional limitations may experience difficulty in transitioning from paediatric to adult healthcare services because of differences in services and reduced support from healthcare professionals that occur during the transition (Castillo & Kitsos 2017). For this reason, a structured HCT process is necessary.

The process of HCT bridges the gap between supervised, family-centred paediatric-oriented care, to an adult-centred model in which the adolescent has increased autonomy. This process may or may not involve a change in healthcare provider. The success of the transition is vital for the optimal, long-term health of adolescents (Gabriel et al. 2017) and is therefore a staggered process. Healthcare transition is a long-term process that includes three main phases: transition preparation or planning, transfer from paediatric to adult-oriented care and integration into adult care (White & Cooley 2018). Healthcare transition is initiated at different times for different individuals and progresses in accordance with the adolescent’s context, level of development, health condition and personal characteristics (Castillo & Kitsos 2017; Hasegawa & Gleeson 2018; Hobart & Phan 2019). Healthcare transition is also an active process where healthcare professionals should continuously collaborate and negotiate with adolescents, caregivers and other healthcare professionals to provide appropriate support and health services throughout the HCT process (White & Cooley 2018).

Emerging studies on HCT indicate many challenges experienced throughout the process, both globally and in Africa (Abaka & Nutor 2021; Betz et al. 2013; Kung et al. 2016; Mbalinda et al. 2021; Westwood, Langerak & Fieggen 2014; Zanoni et al. 2020). In Africa, some studies indicate that the transition of many adolescents to adult-oriented care remains an age-related abrupt shift with adolescents feeling insufficiently prepared for the demands of adult-oriented healthcare (Abaka & Nutor 2021; Mbalinda et al. 2021; Westwood et al. 2014; Zanoni et al. 2020). Other studies indicate that clinicians have begun to apply more stringent clinical reasoning to the transition process, including identifying transition readiness and providing counselling and education (Haghighat et al. 2019; Zanoni et al. 2021).

Limited research is available on current HCT practices used in occupational therapy when transitioning service-users from paediatric- to adult-oriented care. Existing HCT models and guidelines have been developed in other health fields, predominantly in medicine (Hobart & Phan 2019; Kerin, Lynch & McNicholas 2020; Pierce, Hossain & Gannon 2020; Ritchwood et al. 2020). The role of occupational therapy in HCT, as well as current occupational therapy HCT practices and challenges, is not well documented. Occupational therapy plays a vital role in facilitating the functional independence of adolescents with special health needs as they transition from childhood roles to adult roles, thereby ensuring they are able to make a meaningful contribution to their society (Clarkson, Boshoff & Kernot 2021; Levanon-Erez et al. 2017; Toska et al. 2019). Occupational therapy also has a significant role to play in fostering the development of autonomy and self-management skills in adolescents with physical or intellectual disabilities, so that they are better able to manage their health needs as they enter adulthood (American Occupational Therapy Association 2020). Occupational therapists are thus well positioned to coordinate the HCT process and ensure adolescents are adequately prepared for meeting the demands of adult roles, including health management. For this reason, the development of an effective HCT process is important in the management of adolescents within occupational therapy services.

This study aims to discuss current HCT perceptions and practices within South African occupational therapy services and evaluate these against HCT literature and best practice guidelines. This process is essential to ensure consistent delivery of quality health services (Esposito 2014).

## Research methods and design

### Study design

The study follows a quantitative, non-experimental and descriptive cross-sectional survey design (Cresswell & Cresswell 2018).

### Population and sampling

The research population consisted of occupational therapists currently working in South African public health facilities. Public healthcare in South Africa is government-funded, available to all citizens and utilised by the majority (83.8%) of the population (Maphumulo & Bhengu 2019). The public healthcare system has three levels of healthcare: tertiary specialised hospitals located in major cities, secondary provincial and district hospitals, and local primary healthcare centres (Maphumulo & Bhengu 2019).

The number of occupational therapists currently involved in HCT within South African public health facilities is unknown because of the absence of prior studies. Hence, the full cohort of occupational therapists in public health facilities were invited to participate in the study. The total number of occupational therapists in independent practice within the public healthcare sector was estimated at 1220 using available data (Health Professions Council of South Africa [HPCSA] 2021; Ned, Cloete & Mji 2017; Occupational Therapy Association of South Africa [OTASA] 2021).

A combination of simple convenience and snowball sampling was used to recruit participants via the OTASA, Rural Rehab South Africa (RURESA), the ‘OT Tree’ - a local email network- and social media-based occupational therapy forums.

### Inclusion criteria and sample composition

The inclusion criteria were occupational therapists currently working in South African public health facilities, registered with the HPCSA as independent practitioners, with a minimum 1 year of working experience in a public health facility.

According to the HPCSA annual report 2020/2021, 5876 occupational therapists were registered with the HPCSA in 2021 (HPCSA 2021). Of these, 25.2% were expected to be practising in public healthcare (Ned et al. 2020). Using these figures, around 1480 occupational therapists can be identified as practising in public healthcare. As the number of public healthcare posts tends to remain the same or decrease because of the freezing of posts (Ned et al. 2020), it is assumed that the number of occupational therapists in public hospitals would not have increased substantially since 2021. By excluding community service occupational therapists, who are approximately 260 graduates per year (World Federation of Occupational Therapists 2020), the total number of occupational therapists in independent practice within public healthcare can be estimated at 1220. Many registered professionals may not be practising in South Africa currently as they may have emigrated abroad but elected to retain their HPCSA membership (Ned et al. 2020). This may mean that the actual population of actively practising South African occupational therapists is significantly lower. According to Cochran’s sample size formula for categorical data, with an estimated maximum population of 1220 occupational therapists working in public hospitals in South Africa, an alpha level of 0.025 and a margin of error of 0.05, the estimated minimum returned sample size was 278 participants (Bartlett, Kotrlik & Higgins 2001).

### Data collection

Data were collected through an online survey administered using Research Electronic Data Capture (REDCap^®^), an electronic data capture tool (Harris et al. 2009, 2019). The steps outlined by Polit and Yang (2016), as well as existing literature, were used in the design of the survey. The survey was piloted with five occupational therapists who met the inclusion criteria of the study and had over 10 years of experience in a public healthcare facility. Based on the content validity scores, the survey was adapted for gathering valid data.

The finalised survey was administered online via REDCap^®^ (Harris et al. 2009, 2019) between January and July 2023. Participants were able to access the survey questionnaire using a digital link that was propagated through the OTASA, RuReSA and OT Tree networks, as well as through occupational therapy social media groups.

The *Protection of Private Information Act* and the HPCSA General Ethical Guidelines for Health Researchers guided the management of data (HPCSA 2016; South African Government 2013). Survey responses were anonymous and collected no identifiable information. Data were collected and stored electronically via REDCap^®^, which is an online, access-controlled cloud-based platform hosted by the university, which provided ethical approval. Raw data were not stored on any hard drives because of the risk of loss, theft or damage.

### Data analysis

Simple descriptive statistics methods were used to analyse the information. Conventional content analysis was used to analyse open-ended questions. Answers were categorised and represented in terms of the frequency of identified themes. All data were represented graphically and thereafter analysed in relation to existing literature and published best practice guidelines on HCT (American Occupational Therapy Association 2018; HPCSA 2020; World Health Organization 2020).

### Rigour of the study

The accuracy of the self-developed survey was assessed by piloting the instrument to establish content validity (Cresswell & Cresswell 2018; Polit & Yang 2016), as well as through using literature as the basis for the development of the tool. The content validity scores strengthened the validity of the final questionnaire. The guidelines outlined by Polit, Beck and Owen (2007) were used to determine criteria for retention, revision or elimination based on item-level content validity index (I-CVI) scores. Items with an I-CVI score of below 0.99 were revised, but as none were below 0.78, no items were eliminated based on I-CVI scores. The scale-level content validity index (S-CVI/Ave) was 0.99, which is interpreted as excellent (Polit et al. 2007). Because there was a potential for social desirability response bias within this study, the anonymity of survey participants was used to encourage candid responses (Polit & Yang 2016).

### Ethical considerations

Ethical clearance was obtained from the University of the Witwatersrand, Human Research Ethics Committee (HREC)(No. M211054). The following ethical principles outlined by the HPCSA were adhered to during the research process (HPCSA 2016): informed consent, autonomy, confidentiality and non-maleficence. An information letter was attached to the digital invitations and consent was obtained at the start of the survey. Participants could exit the survey at any time without negative consequences. As the survey was distributed via emails and social media, participation was voluntary and accessible at a time convenient to the participants. No information that could identify participants was collected. No risk was anticipated for the participants as a result of their participation in this study. Participants derived no direct benefits from participation in this study.

## Results

In this study, the results have been presented according to the four sections of the survey:

- Section 1: Demographic profile
- Section 2: Healthcare professionals’ understanding of the HCT process
- Section 3: HCT practices of occupational therapists
- Section 4: Factors that promote or hinder the success of HCT within occupational therapy services in South African public health facilities.

A total of 74 respondents consented to participate in the survey. During the process of data cleaning, a total of nine responses were discarded. Eight of these responses did not meet the inclusion criteria, while one response was discarded because of being a duplicate. The remaining 65 responses were utilised for the study, equating to a response rate of 5.3%. This response rate is within the range of 3.6% to 13% reported by previous researchers surveying a similar population (Monareng, Franzsen & Van Biljon 2018; Pitout 2014; Ver Loren Van Themaat 2015).

**FIGURE 1:**
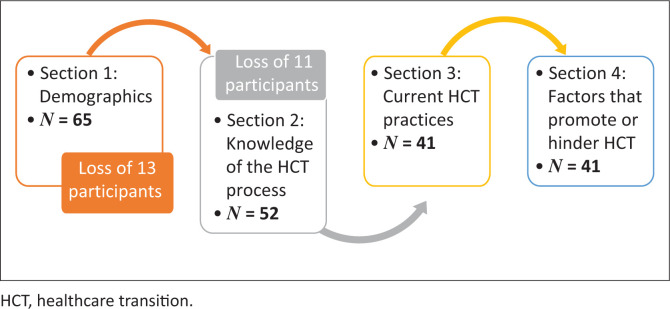
Number of participant responses per section.

Figure 1 details the number of responses obtained per section. Sixty-five participants completed Section 1, 52 participants completed Section 2 and 41 participants completed Sections 3 and 4. The loss of 24 participants between Section 1 and Section 4 of the survey may be because of survey fatigue or a lack of familiarity with the topic. Notably, of the 24 participants who withdrew during the survey, 15 indicated that they worked with service-users across the lifespan, which may indicate that occupational therapists do not perceive a need to implement HCT processes where there is no change in healthcare professional between childhood and adulthood.

### Section 1: Demographic profile

The first section of the study provided a profile of participants involved in the study.

As can be seen in Table 1, the majority of participants had less than 10 years’ experience in public healthcare, with the highest frequency present in the category of 1–5 years’ experience. Representation was received from all levels of public health services and eight out of nine provinces.

**TABLE 1:** Demographic profile of occupational therapists who participated in the study (*N* = 65).

Variable	Category	Frequency (*N* = 65)	Percentage (%)
Years of experience in public healthcare	1–5 years	25	38.5
	6–10 years	22	33.9
	> 10 years	18	27.7
Type of public health facility	Tertiary (academic) hospital	26	40.0
	District hospital	21	32.3
	Central (academic) hospital	6	9.2
	Regional or provincial hospital	5	7.7
	Other (state-owned enterprises, specialised psychiatric or rehabilitation facilities)	5	7.7
	Primary healthcare facility	2	3.1
Age range of service-users seen	0 years and above (full age range)	23	35.4
	0–12 years	10	15.4
	0–18 years	2	3.1
	13–18 years	3	4.6
	> 13 years	24	36.9
	None selected	3	4.6
Conditions seen	Neurological disorders	51	78.5
	Orthopaedic conditions	37	56.9
	Learning disabilities	36	55.4
	Congenital disorder	33	50.8
	Psychiatric disorders	25	38.5
	Chronic illness	22	33.9
	Sensory integration difficulties	22	33.9
	Visual impairment	21	32.3
	Other (e.g. burns)	5	7.7
Role in HCT	Working with service-users along the full child-to-adult age spectrum	33	50.8
	Referring to adult-oriented occupational therapy services	28	43.1
	Receiving service-users from child-oriented occupational therapy	18	27.7
	None selected	3	4.6

HCT, healthcare transition.

The age range of service-users seen varied among participants. Participants were able to select more than one category, as some participants saw multiple age categories at once, or rotated within the department, seeing different age categories at different times. A large proportion (35.4%) of participants worked with service-users across the full age range. The distribution of service-users by age suggests that service-users between the ages of 13 and 18 are more commonly seen by therapists who provide adult-orientated services (36.9%), as opposed to child-orientated services (15.4%) or purely adolescent services (4.6%).

Participants reported working with a wide variety of conditions in the adult and paediatric population in public health facilities. The most frequently seen conditions were neurological disorders (78.5%), orthopaedic conditions (56.9%), learning disabilities (55.4%) and congenital disorders (50.8%). Other conditions that were commonly encountered were psychiatric disorders (38.5%), chronic medical illnesses (33.9%), sensory integration difficulties (33.9%) and visual impairment (32.3%). A small proportion of therapists reported working in specific fields, such as burns, functional capacity evaluations and medicolegal cases (7.7%).

The majority of participants (50.8%) provided both child- and adult-oriented services within the scope of their practice. Where a clear demarcation existed between adult and paediatric services, 43.1% referred service-users to adult-oriented occupational therapy and 27.7% received adolescent referrals from paediatric-oriented therapists. This question allowed participants to select more than one option. Where multiple options were selected, the participants reported working in a rotational role, where therapists spent a fraction of each year in different occupational therapy sections within the department, such as adult orthopaedic or neurological rehabilitation, paediatric rehabilitation or psychiatric rehabilitation. Some of these sections worked with service-users across the full age range with a specific condition, such as orthopaedic conditions, while other sections, such as paediatrics, were clearly demarcated along age lines.

### Section 2: Healthcare professionals’ understanding of the healthcare transition process

**FIGURE 2:**
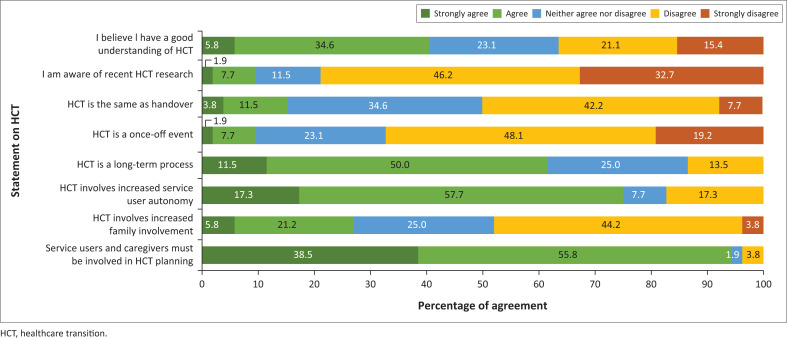
Occupational therapists’ awareness and knowledge of healthcare transition (*N* = 52).

The second section investigated general perceptions held by occupational therapists around HCT using a series of Likert scale questions. Figure 2 presents a visual representation of these perceptions.

#### Self-perceived awareness and knowledge of healthcare transition

As can be seen in Figure 2, 40.4% of participants believed they had a good understanding of HCT (agree or strongly agree), while 36.5% expressed disagreement with the statement. In a follow-up question, participants reported deriving the majority of their understanding of HCT from in-service experience (51.9%). Other sources of knowledge on HCT were undergraduate training (15.4%), continuing professional development (CPD) programmes (7.7%), publications (7.7%) and postgraduate training (3.9%). Almost 10% of participants indicated that they were aware of recent research on HCT, with 78.8% expressing disagreement with this statement.

#### Understanding of core healthcare transition concepts

The majority of participants expressed disagreement with the statements that HCT is the same as handover (50%) and that HCT is a once-off event (67.3%); however, a large proportion of participants expressed a neutral response to these statements. The majority of participants agreed that HCT is a long-term process (61.5%) and that it involves increased autonomy of the service-user (75%). Responses around the degree of family involvement during HCT varied, with 48.1% of participants believing that family involvement should decrease during HCT and 26.9% believing that family involvement should increase during HCT. Participants expressed a strong agreement (94%) that service-users and caregivers should be involved in HCT planning.

**FIGURE 3:**
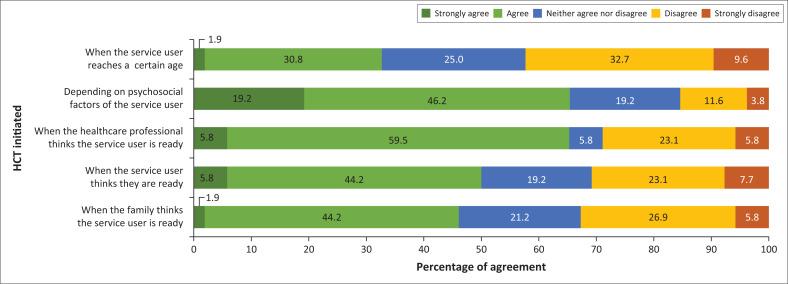
Occupational therapists’ perception of factors that should influence the timing of transition (*N* = 52).

In order of participant agreement, the factors that participants felt should influence when HCT commences as shown in Figure 3 are as follows: psychosocial factors (such as level of maturity, family situation, schooling etc. with 65.4% agreement), healthcare professionals’ determination of readiness through clinical reasoning (65.4%), service-users subjective perception of their readiness (50%), the family’s subjective perception of the service-user’s readiness (46.1%), and the age of the service-user (32.7%).

### Section 3: Healthcare transition practices of occupational therapists

This section reports the main HCT practices of occupational therapists in South African public health facilities, focussing on departmental HCT policies and guidelines, preparation for transition, stakeholder collaboration and outcome measurement.

Table 2 indicates the practices used by occupational therapists within South African public health facilities when implementing HCT.

**TABLE 2:** Current healthcare transition practices of occupational therapists in South African public health facilities.

Variable	Category	Freq. (*N* = 41)	Percentage (%)
Policies used to guide the HCT process	None	12	29.3
	Verbal or written policy indicating the age of transfer	17	41.5
	Written policy on the process of HCT	7	17.1
	Written policy on the HCT process, with input from youth and caregivers	1	2.4
	Other (no department to transfer to)	4	9.8
Planning and preparation for transition	There is no transition plan	17	41.5
	Referral done to adult-oriented therapist	11	26.8
	Goal-directed plan of care created (with or without referral to new therapist)	3	7.3
	Goal-directed plan of care created with service-user and caregiver (with or without referral to new therapist)	5	12.2
	Other	5	12.2
When is a service-user deemed ready for a transition?	At a stipulated age	27	65.9
	Subjectively determined by referring therapist	6	14.6
	Objectively evaluated using a transition evaluation	1	2.4
	Objectively evaluated using a standardised transition evaluation	2	4.9
	Other	5	12.2
Collaboration between paediatric- and adult-oriented therapists	Service-user’s records sent to new therapist	19	46.3
	Service-user’s records, and report or summary sent to a new therapist	5	12.2
	Transfer package sent to the new therapist	2	4.9
	Transfer package sent to the new therapist and direct communication with new therapist	2	4.9
	No change in therapist occurs	9	22.0
	Other	4	9.8
Service-user and caregiver involvement in reviewing the HCT process (policies, guidelines and evaluations)	No involvement	26	63.4
	Some involvement	3	7.3
	Consistent involvement	2	4.8
	Consistent involvement and service-users and caregivers involved in creating, planning, reviewing and disseminating HCT resources	5	12.2
	Other	5	12.2
Measuring outcomes: How is feedback obtained on the HCT process?	No formal process	27	65.9
	Informal verbal feedback sessions used to evaluate the HCT process	10	24.4
	HCT feedback survey is used	0	0.0
	HCT feedback survey is used, and service-users and caregivers involved in survey development and review	1	2.4
	Other	3	7.3

HCT, healthcare transition; Freq., Frequency of engagement in HCT tasks.

#### Policies used to guide the healthcare transition process

Twenty-nine per cent of participants indicated the absence of a departmental policy on HCT. Where HCT policies existed, 41.5% of participants indicated that policy stipulated just the age of transfer, while 17.1% of participants had an HCT policy that additionally described the process that should be followed when transitioning a service-user from paediatric to adult services.

The majority of participants were either somewhat familiar (34.1%) or unfamiliar (29.3%) with their departmental policies on HCT, with only 17.1% of participants expressing good familiarity with departmental HCT policies (extremely familiar: 7.3% and very familiar: 9.8%).

#### Planning and preparation for transition

This section investigated the processes used to identify service-users in need of transition to adult care and prepare service-users for the transition process. A significant proportion of participants (41.5%) indicated that there was no formal plan drawn up to prepare the service-user for the transition to adult-oriented services. For 26.8% of participants, a referral to adult services was the extent of transition planning.

In a follow-up question, participants reported that changes in privacy were not always discussed with the service-user or caregiver in preparation for transition. Where the discussion around privacy took place, more participants reported routinely discussing changes in privacy with the parent (36.6%) than the service-user (22%).

Five of the 41 participants who completed this section of the survey selected ‘other’ to all questions in this section, stating in the comments that these preparatory processes were not applicable to their departments because of a lack of any HCT services within their practice context.

### Determining transition readiness

The majority of participants (58.5%) identified service-users in need of transition on an individual basis. The initiation of transition appears to be primarily based on age, as 65.9% of participants reported initiating transition at a stipulated age. A subjective evaluation of readiness by the referring therapist was used by 14.6% of respondents. While 7.3% of participants indicated that an objective transition readiness evaluation was used, no transition evaluations were named in the comments section provided. Instead, other evaluations, such as literacy tests, were named. It is likely that participants are unaware of HCT models and tools, possibly because of limited familiarity with current research on HCT.

#### Collaboration between paediatric- and adult-oriented therapists

Twenty-two per cent of participants indicated that no change in therapist occurred, as the same therapist provided both child- and adult-oriented services. Where a change in therapist occurred, 46.3% of participants communicated with the new therapist by sending the service-user’s records and/or patient file to the new therapist. Fewer than 10% of participants made use of a comprehensive transfer package containing therapy records, reports or summaries, goals, action plans, transition readiness evaluations and legal documents.

#### Service-user and caregiver involvement in reviewing the healthcare transition process

A significant percentage of participants (63.4%) report no involvement of service-users and caregivers throughout the HCT process. A number of participants (12.2%) reported consistent involvement of service-users and caregivers in creating, planning, reviewing and disseminating HCT resources.

#### Measuring outcomes of the healthcare transition process

The majority of participants (65.9%) do not use any formal process to measure the success of the HCT process. Informal verbal feedback sessions are used by 24.4% of participants to evaluate the HCT process. When rating the perceived effectiveness of their current HCT processes in a follow-up question, the majority of participants (63.5%) rated the HCT process as either ineffective (34.2%) or minimally effective (29.3%). Departmental HCT processes were rated as somewhat effective by 29.3% of participants and very effective by 7.3% of participants.

An open-ended question allowed participants to state what could improve the effectiveness of current HCT practices. A content analysis of the responses revealed a high frequency of participants (46.3%) citing the need for education and training on HCT as a means of improving HCT practice, as many had not been exposed to the concept previously, or felt they had a limited understanding of HCT practice. A total of 24.4% of participants stated that policies and guidelines on HCT needed to be put into place to guide the HCT process. About 9.8% of participants stated that communication between different stakeholders in the HCT process needed to be improved. Other recommendations made by participants included the need for improving the resources required for HCT, such as more permanent staff members, adolescent wards and programmes, and better record-keeping systems so that the outcomes of HCT may be more accurately measured.

### Section 4: Factors that promote or hinder the success of healthcare transition within occupational therapy services in South African public health facilities

**FIGURE 4:**
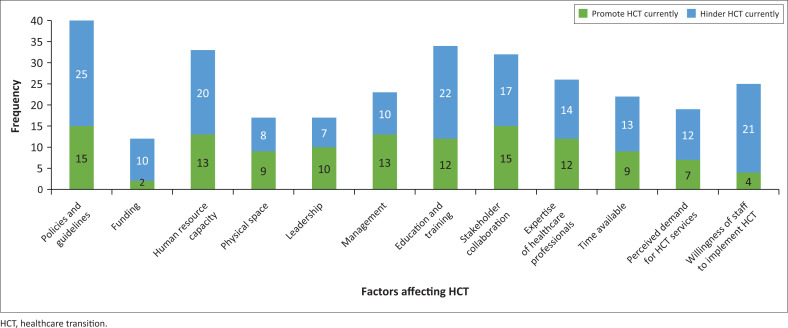
Factors perceived to promote or hinder healthcare transition implementation by occupational therapists in South African public health facilities (*N* = 41).

In the survey, participants were provided with a list of factors that could be selected as a facilitator, and/or a barrier, or neither, to HCT practices. For example ‘policies & guidelines’ may have been selected as a facilitator by participants with established HCT guidelines in the workplace, whereas a participant with a lack of HCT policies and guidelines could have selected this factor as a barrier. Given that the list was not collectively exhaustive, an ‘other’ option was available for text responses. The duality of each factor as either a promoter or facilitator is presented in Figure 4 in terms of frequencies. Overall, more factors were perceived as hindrances than promoters.

As shown in Figure 4, the three main factors perceived by participants as hindering the HCT process in the workplace are insufficient development of policies and guidelines (61%, *n* = 25), inadequate education and training (53.7%, *n* = 22) and limited willingness and motivation to carry out HCT programmes (51.2%, *n* = 21). The three greatest factors identified by participants (*n* = 41) as promoting HCT processes are the presence of policies and guidelines (36.6%, *n* = 15), stakeholder collaboration (36.6%, *n* = 15) and human resource capacity (31.7%, *n* = 13) indicating that sufficient therapists were present to support the implementation of HCT. Effective management at a departmental and institutional level was also cited as an important factor in promoting HCT by 31.7% (*n* = 13) of participants.

## Discussion

### Demographic profile of participants

Responses were received from participants across all levels of South African public healthcare facilities, working with service-users with a variety of conditions such as physical and/or intellectual disabilities, psychiatric disorders and chronic illnesses. This study provided a broad overview of occupational therapists’ HCT practices within the South African public health system. The majority of participants had less than 10 years’ experience in public healthcare, with the highest frequency present in the category of 1–5 years’ experience. The higher prevalence of newer therapists in this study is consistent with findings by Ned et al. (2020), who found that retention of occupational therapists in the public health system is poor, resulting in a high therapist turnover and a younger workforce. Considering that most participants derived their knowledge of HCT from colleagues through in-service experience, occupational therapy departments in public health facilities may have limited knowledge-sharing and execution of HCT practices.

### Perceptions and knowledge of healthcare transition

Participants’ limited knowledge of HCT was identified within this study. While many participants initially perceived themselves to have a good understanding of HCT, later responses revealed misconceptions about HCT concepts and an awareness by participants that education and training on HCT is needed. Previous studies conducted in the United States of America have yielded similar findings: many healthcare professionals have reported feeling unprepared for engaging in the HCT process because of a lack of training and expertise in HCT processes (Anderson et al. 2018; Sadun et al. 2019).

Some core concepts of HCT resonated with participants. Most participants agreed that HCT is not a once-off event. This is in line with the Society for Adolescent Medicine’s definition of HCT, which describes it as a long-term process occurring between childhood and adolescence (Blum et al. 1993). Participants also agreed that HCT should commence when the service-user is ready, which demonstrates an awareness of the need for assessing transition readiness. Considering the individualised personal and health needs of the adolescent before initiating transition is one of the key factors influencing the success of the HCT process (Ritchwood et al. 2020; Tepper, Zaner & Ryscavage 2017).

A significant misconception that emerged at various points throughout the responses is the perception that HCT is not necessary if the service-user remains with the same therapist during their transition from childhood to adulthood. This emerged in the content analysis of the open-ended questions, as participants cited no change in the therapist as a potential reason why HCT, in particular, transition readiness and planning, was not needed. Healthcare transition is still considered necessary even with no change in therapist, as there is still a need to move the adolescent from the more family-orientated, supervised services of paediatric-oriented care, to the individual-focussed adult-oriented care that grants the service-user more autonomy as they begin to navigate adult roles and vocation (Castillo & Kitsos 2017; Robertson 2006).

### Current practices of occupational therapists in South African public health facilities

Within this study, the reported HCT practices of occupational therapists within South African public health facilities vary. Policy on HCT, where it exists, is often limited and HCT tools, such as transition readiness questionnaires or outcome measures, do not appear to be routinely used. Limited policies and guidelines are likely to affect the standardisation of HCT processes and measurement of HCT outcomes within this context.

#### Discrepancy between the perception of healthcare transition and current practice

Although an understanding of some HCT concepts was expressed by participants, this did not appear to translate into routine practice. While participants selected the age of the service-user as the least important factor in determining transition readiness, 66% of participants used age as the criterion for initiating transition within their practice settings. Although 94% of participants agreed that service-users and caregivers should be involved in the HCT process, 63.4% reported no input from caregivers and service-users in current HCT practice.

Occupational therapy as a profession stresses the importance of client-centred practice in collaboration with the service-user and their family (Kielhofner 2009). This value is an integral part of the contemporary paradigm of the profession and forms an important aspect of undergraduate occupational therapy training. Family and client-centred practice is also recognised as an important core competency within the WHO Rehabilitation competency framework (World Health Organization 2020). These underlying professional principles may have guided participants to identify the discrepancy between what HCT should look like and current HCT practice, with 63.5% of participants evaluating their department’s HCT practices as ineffective or minimally effective. The discrepancy between the values of the occupational therapy profession and current HCT practices may result from an ineffective translation of occupational therapy values into the public health system. A previous study conducted on South African occupational therapists identified a similar tendency for occupational therapists working in public healthcare to fall into a mechanistic or medical model of practice that sometimes fails to embody the fundamental philosophy of the profession (Naidoo, Van Wyk & Joubert 2016).

#### Current healthcare transition practice pathways

It appears that current HCT practices among occupational therapists in South African public health facilities take two possible pathways: (1) service-users remain with the same occupational therapist throughout the therapy process until discharge or (2) service-users are referred from paediatric to adult-oriented occupational therapists when deemed ready.

**Pathway 1: Service-users remain with the same occupational therapist throughout the therapy process:** This study found that half the participating public sector occupational therapists (50.1%) work with service-users across the lifespan. This is as a result of either being the only therapist in the department or because of a rotational system in which therapists see service-users according to their availability. As mentioned previously, the lack of change in therapists was perceived by some participants as a reason why HCT processes were considered unnecessary. This indicates a need for clarifying the process of HCT in cases where there is no change in healthcare professionals, as this appears to be a significant therapy context within South African public health facilities.

According to the Got Transition guidelines, the HCT process without change in healthcare professionals would still require the healthcare professional to assess the service-user’s readiness for transition to an adult model of healthcare and prepare the service-user to assume greater autonomy for managing their healthcare (Got Transition n.d.). Identifying transition readiness and creating a transition plan should be carried out in collaboration with the service-user, family or caregiver and in accordance with the adolescent’s level of development (Castillo & Kitsos 2017; Hasegawa & Gleeson 2018; Hobart & Phan 2019). Some adolescents may be able to work towards setting their own appointments and making decisions about the goals of therapy, while others may require parent or caregiver involvement for a longer period or indefinitely. As a child moves into adolescence and early adulthood, it is expected that the focus of occupational therapy would move from a focus on childhood occupations, such as play, learning and activities of daily living, to adult occupations, such as work, leisure and instrumental activities of daily living (American Occupational Therapy Association 2020). Additionally, it would be expected that service-users begin to assume greater responsibility for their therapy (Castillo & Kitsos 2017). Applying an HCT model would ensure that the therapist prepares both the service-user and family adequately for these changes during adolescence, even where no change in the therapist occurs.

An important part of the preparation process is discussing changes in privacy and confidentiality that accompany the transition from child- to adult-oriented services (Bailey, O’Connell & Pearce 2003; Mbalinda et al. 2021). This process is necessary even when no change in therapist occurs. This study revealed that changes in privacy and confidentiality are not routinely discussed with service-users and caregivers. This indicates a potentially significant gap in the HCT preparation process, which may have ethical implications. Practical training is needed on the ethics involved in the shift from paediatric to adult-oriented services within South African occupational therapy.

**Pathway 2: Service-users are referred from paediatric to adult-oriented occupational therapists when deemed ready:** Where occupational therapy services were split into paediatric-oriented and adult-oriented services, the majority of participants initiated transition at a stipulated age, around the age of 12 to 13 years. The tendency to initiate HCT based on age alone has been documented in HIV-related studies on HCT in South Africa (Westwood et al. 2014; Zanoni et al. 2021). Adolescents appear to be transferred abruptly to adult care at the age of 12 without preparation for the transfer, which has been associated with poor clinical outcomes and reduced retention in care (Zanoni et al. 2020). According to the Society for Adolescent Medicine, the age of the adolescent should not be the primary criterion for initiating transition; instead, transition readiness should be determined by the adolescent’s ability to begin managing their own healthcare (Santelli et al. 2003). While transition readiness assessments have been identified as a useful tool in deciding when to initiate HCT, these measures are not routinely used in practice (Zanoni et al. 2020). This is consistent with the findings of this study.

Within this study, it appears that adolescents from the age of 13 and above were more likely to receive adult-oriented occupational therapy services than paediatric or adolescent-specific services. An abrupt shift to adult-oriented care at the age of 13 may result in adolescents being placed within a model of care that they are unprepared for or that does not meet their developmental needs. Adult-oriented health services tend to be characterised by a significant reduction in support by a healthcare professional and an increased demand for service-users to take responsibility for their own health (Ritchwood et al. 2020). The support of healthcare providers during HCT is a key factor in adolescents’ experience of the HCT process in South Africa (Malapela, Thupayagale-Tshweneagae & Ibitoye 2020). The lack of support and perceived inability to meet the demands of adult care has been associated with a reduction in adherence to healthcare services and a tendency to miss appointments (Abaka & Nutor 2021; Mbalinda et al. 2021; Overbury et al. 2021; Wan et al. 2019). Inadequate therapist support during HCT poses a significant risk that adolescents may be lost to occupational therapy services at a critical stage in their development.

A lack of access to occupational therapy services in adolescence may result in poor support for adolescents during the development of important life roles and occupations, such as educational activities and social participation. These occupations form the foundation for prevocational and vocational roles in adulthood; hence, the absence of support for successful engagement in these occupations may have a negative effect on the overall functional outcomes of adolescents with disabilities or chronic health needs (Eismann et al. 2017; Weiss et al. 2021).

According to the Occupational Therapy Practice Framework 4th edition, occupational therapy services during adolescence should include preparation for transition, education on the change in roles and expectations during transition, and assistance to adapt to new roles or life situations (American Occupational Therapy Association 2020). The continuity of occupational therapy services during the transition period is critical to improve the overall functional outcomes of adolescents with disabilities or chronic health needs (Eismann et al. 2017; Weiss et al. 2021). This necessitates the presence of an effective HCT process that ensures adolescents continue utilising occupational therapy services until they no longer require these.

### Factors that promote healthcare transition processes

#### Policies and guidelines

The presence or absence of policies and guidelines on HCT was the main factor identified as influencing the HCT process. Fifteen participants viewed the presence of an HCT policy or guideline as a factor that promoted the effective implementation of HCT, stating that it ensured service-users were transitioned rather than being lost. However, this study also found that most participants (41.5%) have HCT policies or guidelines at their workplace that just stipulate the age at which a child is transferred to adult services. This finding is supported by another South African study that found HCT protocols are based on the child’s age and do not appear to outline the process of HCT or recommend available transition evaluations or tools (Zanoni et al. 2020). Although some therapists may perceive current age-based HCT guidelines to be sufficient, therapists may not be aware of the detrimental impact of an abrupt shift to adult services without adequate preparation. It is thus recommended that current HCT policies used by occupational therapists are updated to adequately guide therapists on best practices.

#### Stakeholder collaboration

Good stakeholder collaboration was a factor perceived to promote the effectiveness of the HCT process by 15 participants. In cases where transition was accompanied by a change in therapist, the presence of communication channels, particularly between paediatric- and adult-oriented occupational therapists, was a factor that assisted in ensuring a smoother transition. These communication channels have been identified as one of the most important predictors of successful HCT (Hobart & Phan 2019). Communication between paediatric and adult occupational therapy services is critical for a seamless transition process, as it is important that both healthcare professionals understand the adolescent’s context, history and needs (Kerin et al. 2020; Ritchwood et al. 2020).

Where a change in occupational therapist occurred during adolescence, the majority of participants indicated that communication between paediatric and adult occupational therapy services consists mainly of sending the service-user’s records and/or a report or summary to the new therapist. This suggests the potential for improving communication through additional measures, such as joint sessions and pre-transition meetings involving adolescents and clinicians from both health services (Jones, Ritchwood & Taggart 2019).

Although participants highlighted stakeholder collaboration as a promoter of HCT, not all stakeholders appear to be part of the collaborative process. Despite 94% of participants indicating that service-users and caregivers should be involved in planning the HCT process, input from youth and caregivers was rarely considered when developing HCT policy. The vast majority of participants indicated that there was no involvement of service-users and caregivers in reviewing the HCT process (policies, guidelines and evaluations) to evaluate outcomes. Studies show that consultation with adolescents and parents is often not optimised during the HCT process, which can cause confusion and impede the transition period (Abaka & Nutor 2021; Jonas et al. 2019; Kerin et al. 2020). Poor involvement of adolescents in the HCT process can result in service-users being inadequately prepared for transition, which can have a negative effect on the outcomes of the HCT process and can result in poor adherence to therapy (Price et al. 2019). It is important that collaborative efforts are extended to service-users and caregivers to enhance the HCT process.

#### Human resource capacity

The presence of sufficient human resource capacity was cited as a factor that promoted the HCT process by 13 participants. This finding contrasts other South African studies that identified human resource capacity as a barrier to the overall implementation of health programmes across the public health sector because of a severe shortage of healthcare professionals (Maphumulo & Bhengu 2019; National Department of Health 2017; Ned et al. 2017). Rehabilitation services in particular often face staffing limitations because of the freezing of posts and a lack of resources (Ned et al. 2020). As a promoting factor, sufficient staffing can be expected to support aspects such as stakeholder collaboration and communication in the HCT process.

#### Management and leadership

Interestingly, the categories of management and leadership were the only two categories that more participants highlighted as promoters of HCT than hindrances to the process. The presence of effective institutional and departmental management has the potential to ensure that HCT processes are adequately planned for, implemented and monitored (Hasegawa & Gleeson 2018).

The perception of management and leadership being a factor that promotes HCT programmes is unexpected. Previous literature indicates that the public health sector is generally characterised by poor leadership and management, which has resulted in corruption, lack of performance monitoring and poor health outcomes for people utilising public health facilities (Maphumulo & Bhengu 2019; National Department of Health 2017; Ned et al. 2017). Contrary perceptions by occupational therapists in public health facilities may indicate positive potential for effective HCT implementation in the future.

### Factors that hinder healthcare transition processes

#### A lack of policies and guidelines

The majority of participants viewed the absence or insufficiency of HCT policies as a hindrance to its effective implementation. Although no formal processes were used to measure the success of HCT, most participants subjectively rated their HCT process as ineffective or minimally effective. This perception is supported by Malapela et al. (2020) and Zanoni et al. (2021), who identified that HCT in South Africa is challenged by the lack of evidence-based protocols, which results in a lack of standardisation of care and can negatively affect HCT outcomes. Poor HCT outcomes can lead to failure to follow up, non-adherence to intervention and, consequently, health complications for adolescents (Kung et al. 2016; Westwood et al. 2014; Zanoni et al. 2021).

To be successful, the HCT process requires policies and guidelines that indicate the criteria for determining transition readiness, beyond just age, as well as the process that should be followed and the roles of different stakeholders within the HCT process (Kerr et al. 2018; Narla et al. 2021). An efficient and context-specific method for measuring HCT outcomes should be included in policy development.

Occupational therapy policies on HCT should also consider the profession’s values and contribution to HCT within the multidisciplinary team. This can include a client-centred and collaborative approach to designing the HCT process, identifying transition readiness based on a holistic evaluation of the adolescent and their life roles, and considering the functional outcomes of successful HCT. Occupational therapy services during the transition should include skills training in developmentally appropriate occupations, such as health management, that take place in the natural context of occupation (Cahill & Beisbier 2020).

The development of HCT programmes and policies that will be effective and meet the needs of South African adolescents requires input and collaboration at multiple levels. Hasegawa and Gleeson (2018) recommend that HCT should be part of hospital standard operating protocols, involving input from senior executives, administrative staff, and both paediatric and adult clinics. They recommend that data should be collected at an institutional level to establish the efficacy of the chosen HCT model. This process is important to allow for the development of large-scale funding models that can ensure adequate financial resources and operationalisation of HCT programmes (Anderson et al. 2018; Blum et al. 1993; Hobart & Phan 2019).

#### Insufficient education and training

Participants’ limited knowledge of HCT concepts and processes was highlighted throughout the study as a significant hindrance to the implementation of HCT. The need for training in HCT was the most frequent theme mentioned by participants as a way of improving the effectiveness of their HCT practice. Training in HCT is needed to develop therapist expertise and improve motivation to put effective HCT programmes into place. This finding correlates with earlier research by Zanoni et al. (2021), who identified limited healthcare professional awareness of and training in HCT as a significant barrier to the production and implementation of HCT protocols.

The development of HCT training programmes for occupational therapists within the South African public health context will require the input of clinicians, academic staff, service-users and caregivers. Training should not only be integrated into undergraduate occupational therapy programmes but also needs to be part of in-service education and mentorship programmes. This is particularly important considering that most participants stated that they derived their knowledge of HCT from in-service experience and were unaware of recent HCT research. Training on HCT should include a focus on adolescent health and development (Anderson et al. 2018; Jones et al. 2019). Training should also equip healthcare professionals with expertise in maintaining the confidentiality and safety of adolescents during the transition (Alderman et al. 2019).

#### Limited willingness and motivation to carry out healthcare transition programmes

A significant barrier to HCT implementation is the limited motivation of occupational therapists to carry out HCT programmes. This may be because of unfamiliarity with the process, a lack of clear departmental protocols on HCT or a high workload that does not leave sufficient mental and physical capacity for carrying out HCT programmes.

The majority (72.31%) of participants had less than 10 years of public healthcare experience, which may limit the perceived ability and willingness to implement novel HCT programmes and improve existing programmes. Ned et al. (2017) report a high turnover of therapists in the South African public health sector, resulting in a younger, less experienced workforce. This can have implications for the implementation of new programmes and processes, as continuity of programmes may be more difficult with a fluctuating workforce.

## Limitations

Participants’ limited familiarity with the topic was evident, as a common variable was participants’ lack of knowledge about HCT and insufficient training in HCT. This is likely to have affected the completion of the survey, as there was a 36.9% loss of participants between Sections 1 and 4. Limited familiarity with HCT may have contributed to misunderstandings regarding HCT concepts, which may have affected participants’ ability to select appropriate responses. For example, participants reported using standardised transition evaluation tools, but analysis of the comments revealed the use of other standardised tests and a possible lack of familiarity with standardised transition tools.

A low response rate (5.3%) means that, while similarities in practice may exist, the findings of this study cannot be generalised to the entire population of South African occupational therapists working in the public health system.

## Conclusion

This study provides a baseline overview of current HCT practices within South African occupational therapy practice. While there appears to be an awareness of the need for HCT among participants, challenges exist in the understanding and practical implementation of HCT within the context of South African public occupational therapy services.

There is a need for improved education of clinicians on HCT concepts and guidelines. Training on HCT should be integrated into undergraduate curriculum programmes as well as in-service continuing education programmes. Future research in South Africa and other African countries is needed to investigate the role of occupational therapy and other health professions in HCT. Qualitative methods can be utilised to provide more in-depth and nuanced perspectives on how occupational therapists understand HCT. An important aspect that will need to be investigated further is the difference between paediatric and adult models of occupational therapy, and how to optimise the shift between these models of care. This may assist in the development of training programmes in the future.

Realistic and contextually relevant policies on HCT need to be developed to optimise the transition of adolescents from paediatric to adult models of occupational therapy services. This is critical to ensure that South African occupational therapists are able to provide uninterrupted and developmentally appropriate therapy services that can meet the needs of adolescents with chronic health conditions.
